# A Large Animal Model of Right Ventricular Failure due to Chronic Thromboembolic Pulmonary Hypertension: A Focus on Function

**DOI:** 10.3389/fcvm.2018.00189

**Published:** 2019-01-09

**Authors:** Ashley Mulchrone, Heidi B. Kellihan, Omid Forouzan, Timothy A. Hacker, Melissa L. Bates, Christopher J. Francois, Naomi C. Chesler

**Affiliations:** ^1^Department of Biomedical Engineering, Univeristy of Wisconsin-Madison, Madison, WI, United States; ^2^School of Veterinary Medicine, University of Wisconsin-Madison, Madison, WI, United States; ^3^Department of Medicine, University of Wisconsin-Madison, Madison, WI, United States; ^4^Department of Health and Human Physiology, University of Iowa, Iowa City, IA, United States; ^5^Department of Pediatrics, University of Iowa, Iowa City, IA, United States; ^6^Department of Radiology, University of Wisconsin-Madison, Madison, WI, United States

**Keywords:** pulmonary embolization, pulmonary hemodynamics, right ventricular afterload, effective arterial elastance (E_*a*_), pulmonary vascular resistance (PVR)

## Abstract

Chronic thromboembolic pulmonary hypertension (CTEPH) is a debilitating disease that progresses to right ventricular (RV) failure and death if left untreated. Little is known regarding the progression of RV failure in this disease, greatly limiting effective prognoses, and therapeutic interventions. Large animal models enable the use of clinical techniques and technologies to assess progression and diagnose failure, but the existing large animal models of CTEPH have not been shown to replicate the functional consequences of the RV, i.e., RV failure. Here, we created a canine embolization model of CTEPH utilizing only microsphere injections, and we used a combination of right heart catheterization (RHC), echocardiography (echo), and magnetic resonance imaging (MRI) to quantify RV function. Over the course of several months, CTEPH led to a 6-fold increase in pulmonary vascular resistance (PVR) in four adult, male beagles. As evidenced by decreased cardiac index (0.12 ± 0.01 v. 0.07 ± 0.01 [L/(min^*^kg)]; *p* < 0.05), ejection fraction (0.48 ± 0.02 v. 0.31 ± 0.02; *p* < 0.05), and ventricular-vascular coupling ratio (0.95 ± 0.09 v. 0.45 ± 0.05; *p* < 0.05), as well as decreased tricuspid annular plane systolic excursion (TAPSE) (1.37 ± 0.06 v. 0.86 ± 0.05 [cm]; *p* < 0.05) and increased end-diastolic volume index (2.73 ± 0.06 v. 2.98 ± 0.02 [mL/kg]; *p* < 0.05), the model caused RV failure. The ability of this large animal CTEPH model to replicate the hemodynamic consequences of the human disease suggests that it could be utilized for future studies to gain insight into the pathophysiology of CTEPH development, following further optimization.

## Introduction

Chronic thromboembolic pulmonary hypertension (CTEPH) is a debilitating, fast progressing vascular disease associated with poor prognosis and significant morbidity and mortality (1–3). It is one of the most common and potentially curable subsets of precapillary pulmonary hypertension (PH) (4, 5) and is characterized by the obstruction of the pulmonary vasculature from unresolved, organized thromboemboli. The diagnosis of CTEPH is made by mean pulmonary artery pressures (mPAP) ≥25 mmHg, pulmonary capillary wedge pressures (PCWP) ≤ 15 mmHg at rest, and evidence of thromboemboli by an imaging modality (5, 6). Since patients are often asymptomatic or misdiagnosed, CTEPH is typically advanced at the time of diagnosis (7–9).

The mechanical obstruction of the pulmonary vascular bed in CTEPH increases pulmonary vascular resistance (PVR) and right ventricular (RV) afterload. The RV can adapt to the increased afterload for some time to maintain cardiac output (CO), but without treatment, CO and ejection fraction (EF) typically drop and death ensues (2, 10, 11). There are several critical knowledge gaps in this process, including the mechanistic transition from adaptation to maladaptive remodeling, the functional precursors of failure, and the biological indicators of the failed RV. These knowledge gaps limit the development of effective prognoses and optimized patient care (6).

A common approach to address pathophysiological knowledge gaps is preclinical or animal models of disease. Due to their cost-effectiveness, efficiency, and potential for genetic manipulation, rodents are frequently used. Common techniques for studying venous thrombus generation/resolution or CTEPH include pulmonary artery (PA) ligations (12), balloon occlusions (13, 14), and microsphere injections (3). These models provide limited insight into clinical practice as they generally fail to replicate RV failure. Neto-Neves et al. created a successful CTEPH rat model utilizing microsphere injections in conjunction with a vascular endothelial growth factor (VEGF) receptor tyrosine kinase inhibitor (SU5416; Tocris Bioscience, Bristol, UK) that did demonstrate RV remodeling and RV dysfunction, but only one animal was studied out to heart failure (15). Moreover, findings from small-animal models can be strain-specific with significant inter- and intra-species variation, and/or have accelerated disease progression not consistent with clinical presentation (2, 3, 10).

In contrast, large animal models, which incur increased costs and complexities in housing and care, more closely mimic human physiology and pathophysiology (16). Investigations in large animals can also utilize the same techniques and technologies as clinical studies. While acute embolism models are relatively easy to induce, capturing the hallmark characteristics of clinical CTEPH with RV failure remains elusive (2). Many attempts have been made since the 1980's to develop a reliable model of CTEPH in large-animals including pigs, sheep, dogs, cows, and non-human primates with little success. Common techniques typically include some combination of venous thrombosis, surgical ligations or shunts, balloon occlusions, embolic occlusions with microspheres or tissue adhesive, and thrombolytic or VEGF inhibitors (17–23). However, most studies fail to either measure RV function or the model fails to replicate RV failure. Using a swine model of chronic PH (21, 22), Boulate et al. did demonstrate acute RV failure when also inducing volume overload via saline infusion and iterative acute pulmonary embolization (24), but the chronic PH model relied on proximal obstruction of the right lower-lobe artery via tissue adhesive in conjunction with the PA ligation which exhibited no clinical evidence of RV failure at rest (19). Stam et al. also created a swine model of CTEPH utilizing multiple microsphere injections in conjunction with an endothelial nitric synthase inhibitor to cause endothelial dysfunction (25). This model demonstrated decreased cardiac index (CI), RV remodeling, and exercise intolerance, but required open-chested procedures as well as a two-hit mechanism to induce hemodynamic changes.

Here, we sought to create a canine model of CTEPH that could be developed using less invasive surgical procedures, as well as utilize only microsphere injections. We utilized a similar approach as Hori et al., but followed animals until RV failure occurred as evidenced by clinically used invasive and non-invasive metrics such as CO, EF, and end-diastolic volume (EDV) (26). By defining the phenotype and timing of RV failure, we offer a clinically relevant CTEPH model as a tool for studying the mechanism of PH-associated RV remodeling and failure.

## Methods

**Figure 1:**
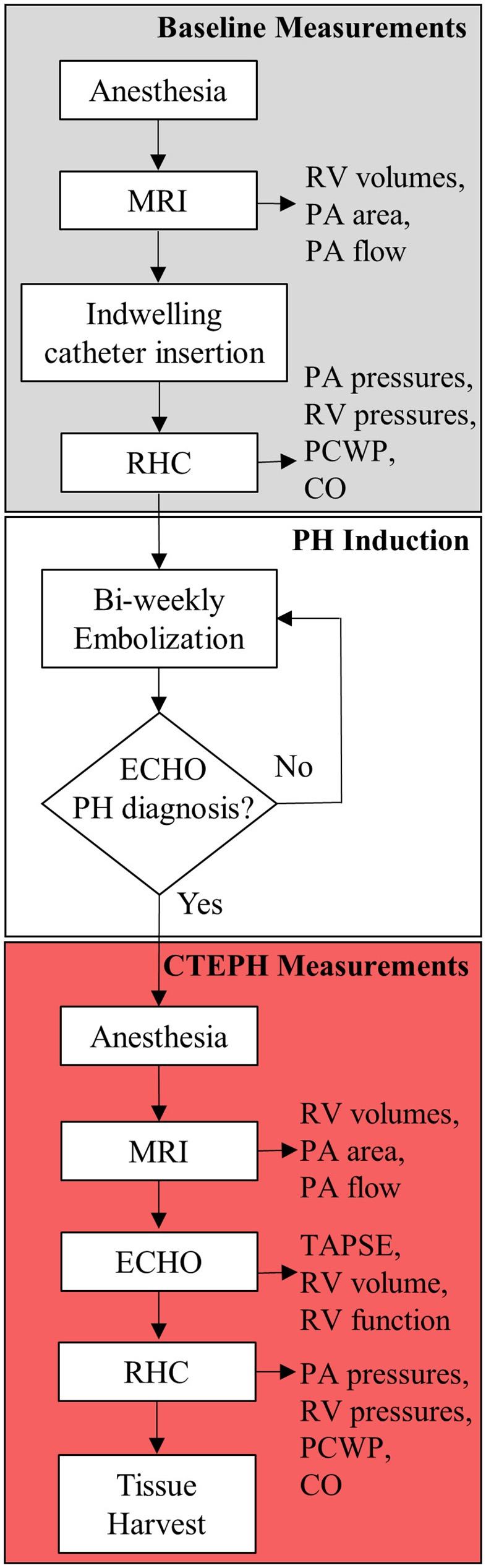
Experimental flowchart highlighting the major experimental procedures.

CTEPH was induced in five, adult male beagles (12 ± 1 kg body weight) following a modified version of an established canine model (26, 27). The protocol is outlined in Figure 1. All procedures were approved by the University of Wisconsin-Madison Institutional Animal Care and Use Committee.

### Induction and Anesthesia

Following pre-medication with hydromorphone (0.1 mg/kg, IM) and midazolam (0.2 mg/kg, IM), general anesthesia was induced by an intravenous (IV) injection of propofol (10 mg/kg). The animals were then intubated, and anesthesia was maintained with isoflurane (1–3%) in 100% oxygen; ventilation was adjusted as needed to maintain appropriate end-tidal CO_2_ levels (30–50 mmHg). Sterile saline was infused via IV access at a rate of 10 mL/(kg^*^h). Cephalexin (30 mg/kg) was given IV. Once stable under anesthesia, the animals were transferred to a magnetic resonance imaging (MRI) suite before returning to the procedure room.

### Magnetic Resonance Imaging

MRI studies were performed on a clinical 3T scanner (MR750, GE Healthcare, Waukesha, WI, USA), using previously reported parameters (27). Briefly, axial ECG-gated CINE balanced steady-state free precession images were acquired through the entire heart. Between 20 and 30-time frames were reconstructed at each slice location (12–25 slices depending on heart size). In addition, two-dimensional phase contrast images were acquired through the main, left, and right PA to assess the relative area change (RAC) and flow.

### Indwelling Catheter Placement and Baseline Hemodynamics

CTEPH was induced by repeated injections of microspheres into the PA via an indwelling catheter. The indwelling catheter was inserted into the femoral vein and advanced into the PA under fluoroscopic guidance with contrast. The distal end was tunneled under the skin and exteriorized at the dorsum between the shoulder blades, where it was sutured in place to prevent movement. A Luer stub was placed on the end of the tubing and a Luer access split septum port was attached.

The femoral artery was catheterized to monitor systemic arterial pressure and arterial blood gases, and the femoral and external jugular veins were catheterized for right heart catheterization (RHC), angiography contrast delivery, and blood sampling. Baseline PA, RV, and right atrial (RA) pressures (mean, systolic, and diastolic) were obtained. CO was measured using thermodilution, in triplicate. Lastly, the indwelling catheter was filled with heparinized saline (1000 USP Units/mL) and the end was taped shut using self-adherent wrap.

### Echocardiography

Transthoracic echocardiography was performed by a board-certified veterinary cardiologist (HBK). Animals were gently restrained in lateral recumbency on a purpose-built table, with small cut-out areas under the thorax. Two-dimensional echocardiography was used to evaluate RV size and function, while color flow and spectral Doppler imaging were performed to assess valve regurgitation. Echocardiographic measurements were also obtained from weight- and sex-matched healthy controls (*n* = 4; body weight = 11 ± 1 kg).

### CTEPH Induction

To induce CTEPH, microspheres were perfused into the pulmonary vasculature every 3–4 days over the course of several months. A 0.5 mL volume of autoclaved 100–300 μm microspheres (Sephadex^TM^ G-50 coarse; GE Healthcare) were vigorously mixed with 20 mL of sterile saline and divided into 2 mL aliquots. Every 3–4 days, 2 mL of this suspension was slowly injected through the access port of the indwelling catheter followed by 2 mL of sterile saline. The access port was replaced with a new or disinfected port as needed. A pressure transducer connected to the port recorded PA pressures. Then, a heparinized solution was added to refill the catheter. Animals were monitored for signs of distress (i.e., respiratory distress, shortness of breath, or collapse) before, during, and following the procedure. To protect catheter integrity, dogs were fitted with surgical jackets and Elizabethan collars, and were housed individually. They were fed a commercial dry food diet and had free access to water. The access port was disinfected with 70% isopropyl alcohol and iodine, and aspirated and replaced with new heparinized saline daily. Microsphere injections were continued until there was evidence of hemodynamically significant PH as determined by the veterinary cardiologist, utilizing echo to monitor progression. Echo measurements of tricuspid regurgitation flow velocity, RV septal flattening, RV concentric hypertrophy, RV dilation, notching of the PA flow profile, and pulmonic regurgitation velocities were performed monthly, at a minimum (28, 29).

### Terminal Procedure

As with the baseline procedure, the animals were pre-medicated with hydromorphone (0.1 mg/kg, IM) and midazolam (0.2 mg/kg, IM), and general anesthesia was induced by an IV injection of propofol (10 mg/kg). Atropine (0.02 mg/kg) was used as needed to stabilize the heart rate. Animals were then intubated, and 0.9% saline was started IV (10 mL/(kg^*^h)). Animals were transferred to the MRI suite where RV and PA structural and flow measurements were measured. The echocardiographic measurements were obtained, and then fluoroscopic guidance was used to insert a pressure catheter where end-point PA and RV pressures were recorded. These pressure measurements were obtained followed by CO measurements, again in triplicate. The CO was corrected for body weight to account for growth, resulting in CI. Lastly, digital subtraction angiography images were acquired to assess changes in lung perfusion (5).

Following the study, the animals were humanely euthanized (5 mL Beuthanasia, IV) and the heart, pulmonary vasculature, and lungs were harvested and preserved for histological analysis and mechanical testing as previously reported (30).

### Data Analysis

The axial ECG-gated CINE balanced steady-state free precession images were used to manually contour the RV in each of the 20–30 time frames for each slice location using Segment software (Medviso, Lund, Sweden). The RV volume was calculated for all phases of the cardiac cycle, and the EDV and end-systolic volume (ESV) were taken as the maximum and minimum reconstructed volume, respectively. Stroke volume (SV) and EF were then calculated as:

(1) $$\begin{array}{rcl} \text{SV} & = & \text{EDV}-\text{ESV} \end{array}$$

(2) $$\begin{array}{rcl} \text{EF} & = & \frac{\text{SV}}{\text{EDV}} \end{array}$$

and a volume-only method was used to estimate the ventricular-vascular coupling (VVC) ratio (27):

(3) $$\begin{array}{r} VVC\;=\;\frac{\text{SV}}{\text{ESV}} \end{array}$$

By combining volumes with recorded pressures, the total arterial compliance, right ventricular stroke work (RVSW), and pulmonary vascular resistance were calculated as:

(4) $$\begin{array}{rcl} \text{Total arterial compliance} & = & \frac{\text{SV}}{\text{sPAP}-\text{dPAP}} \end{array}$$

(5) $$\begin{array}{rcl} \text{RVSW} & = & \left(\text{mPAP}-\text{RAP}\right)*\text{SV} \end{array}$$

(6) $$\begin{array}{rcl} \text{PVR} & = & \frac{\text{mPAP}-\text{PCWP}}{\text{CO}} \end{array}$$

where sPAP and dPAP are the systolic and diastolic pulmonary arterial pressures, respectively, and RAP is the right atrial pressure. The PA cross-sectional area and blood flow were analyzed using the magnitude and phase images of the two-dimensional phase contrast MRI scans, respectively. The cross-sectional area at peak systole (A_max_) and end diastole (A_min_) were then used to calculate the RAC in each of the PAs (31):

(7) $$\begin{array}{rcl} RAC & = & \frac{A_{\max}-A_{\min}}{A_{\max}} \end{array}$$

RAC is a non-invasive measure of proximal arterial stiffening and a predictor of mortality in PH (32). Lastly, a modified version of the Windkessel model was used to estimate the effective arterial elastance (E_a_), a measure of RV afterload (27):

(8) $$E_{a}=\frac{\text{mPAP-PCWP}}{\text{SV}}$$

### Statistical Analysis

All data are reported as the mean ± standard error. The Ryan-Joiner test was used to check for normality. Comparisons between the control and the CTEPH echocardiography data were conducted using a two-sample *t*-test. Comparisons between baseline and CTEPH MRI and RHC data were analyzed using a paired *t*-test. A *p*-value < 0.05 was used to indicate statistical significance. All analyses were conducted on MiniTab® software (PA State College, version 18).

## Results

### CTEPH Induction

**Figure 2:**
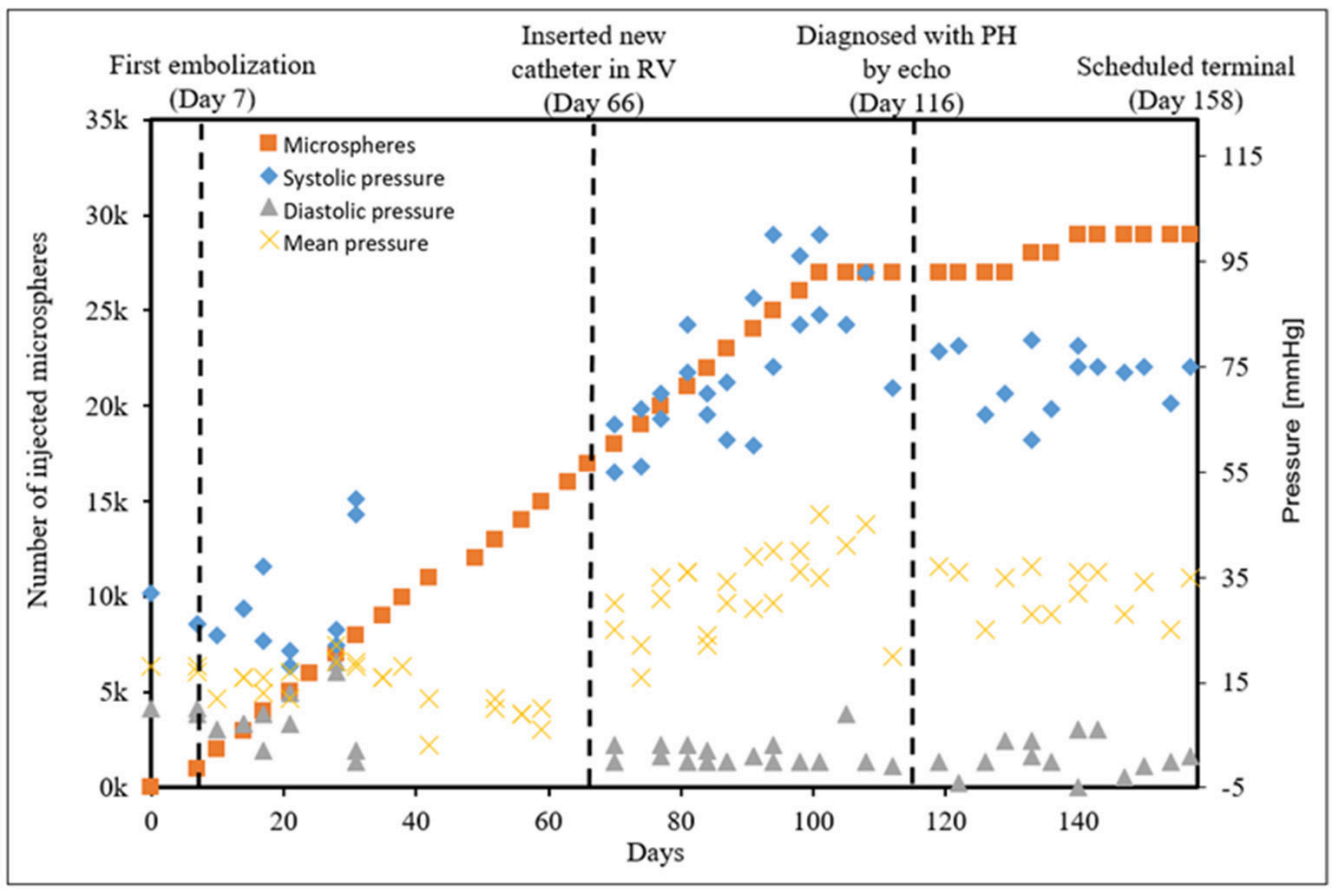
The progressive pressure increases in the main PA obtained from the indwelling catheter over time in a single canine that developed CTEPH.

**Figure 3:**
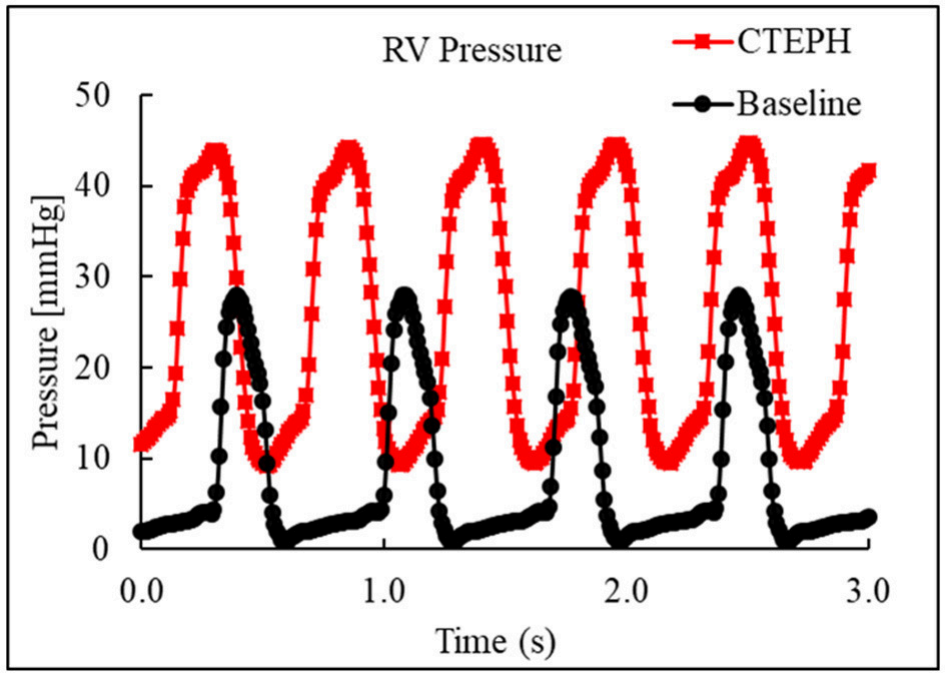
Right ventricular pressure traces from RHC at baseline and at the terminal end-point of CTEPH.

CTEPH was successfully induced in four of the five dogs—the data from one dog was excluded from analysis for failing to meet the requirements for PH diagnosis, mainly insufficient increases in PA pressures. Table 1 summarizes the CTEPH induction times for each canine as well as the estimated number of microspheres. Figure 2 shows the progressive increases in PA pressures obtained from the indwelling catheter, concurrently plotted with the microsphere injections for one of the canines that developed CTEPH, and Figure 3 shows RV pressure traces obtained during RHC at baseline and CTEPH for the same animal. Table 2 summarizes the data obtained from RHC and MRI, and Table 3 contains the data from echo. Overall, chronic embolization caused an increase in PA pressures; mPAP and dPAP doubled (*p* = 0.046 and *p* = 0.036, respectively), while sPAP increased by almost 70% (*p* = 0.106). The PCWP remain unchanged at 10.3 ± 1.3 mmHg (*p* = 1.0).

**Table 1:** Estimated number of microspheres used to induce CTEPH in each canine.

	**PH diagnosis**		**Terminal end-point**	
**Canine**	**# Days**	**# Microspheres**	**# Days**	**# Microspheres**
1	116	27,000	158	29,000
2	115	36,000	199	49,000
3	238	62,000	252	62,000
4	224	61,000	252	65,000

**Table 2:** Data collected from RHC and MRI before and after chronic embolization (*n* = 4).

**Parameter**	**Technique**	**Baseline**	**CTEPH**	***p*-value**
Body weight (kg)	–	12 ± 1	12 ± 1	0.294
Heart rate (bpm)	–	90 ± 2	108 ± 5	0.051
sPAP (mmHg)	RHC	26.5 ± 3.0	44.6 ± 8.3	0.106
dPAP (mmHg)	RHC	11.5 ± 1.2	26.5 ± 5.0	**0.036**
mPAP (mmHg)	RHC	16.5 ± 1.6	34.3 ± 6.0	**0.046**
PCWP (mmHg)	RHC	10.3 ± 0.5	10.3 ± 1.3	1.000
SBP (mmHg)	RHC	–	128 ± 18	–
DBP (mmHg)	RHC	–	72 ± 11	–
MBP (mmHg)	RHC	–	94 ± 13	–
RAP (mmHg)	RHC	6.25 ± 0.95	7.25 ± 1.11	0.630
sRVP (mmHg)	RHC	24.63 ± 3.05	43.50 ± 6.84	0.064
dRVP (mmHg)	RHC	3.75 ± 1.89	5.50 ± 1.32	0.544
mRVP (mmHg)	RHC	12.50 ± 1.26	20.25 ± 3.09	0.072
RV EDV (mL/kg)	MRI	2.73 ± 0.06	2.98 ± 0.02	**0.021**
RV ESV (mL/kg)	MRI	1.41 ± 0.07	2.05 ± 0.06	**0.012**
RV SV (mL/kg)	MRI	1.32 ± 0.07	0.92 ± 0.06	**0.013**

*RHC, right heart catheterization; MRI, magnetic resonance imaging; bpm, beats per minute; sPAP, systolic pulmonary arterial pressure; dPAP, diastolic pulmonary arterial pressure; mPAP, mean pulmonary arterial pressure; PCWP, pulmonary capillary wedge pressure; SBP, systolic blood pressure; DBP, diastolic blood pressure; MBP, mean blood pressure; RAP, right atrial pressure; RV, right ventricle; sRVP, systolic RV pressure; dRVP, diastolic RV pressure; mRVP, mean RV pressure; EDV, end-diastolic volume; ESV, end-systolic volume; SV, stroke volume. Bold indicates p < 0.05*.

**Table 3:** Data collected during echo between CTEPH and healthy controls.

**Parameter**	**Control (*n* = 4)**	**CTEPH (*n* = 4)**	***p*-value**
Body weight (kg)	11 ± 1	12 ± 1	0.531
Heart rate (bpm)	89 ± 11	103 ± 9	0.387
Ao diameter (cm/kg)	0.16 ± 0.01	0.14 ± 0.01	0.340
PA diameter (cm/kg)	0.12 ± 0.01	0.14 ± 0.01	0.078
RV thickness (cm)	0.52 ± 0.07	0.61 ± 0.03	0.352
RV PEP (ms)	35 ± 3	42 ± 6	0.359
RV AT (ms)	87 ± 6	94 ± 13	0.612
RV ET (ms)	211 ± 16	283 ± 14	**0.019**
AT:ET	0.41 ± 0.02	0.33 ± 0.03	0.099
LA diameter (cm/kg)	0.20 ± 0.01	0.17 ± 0.01	0.086
LV mass (g/kg)	6.23 ± 0.55	3.89 ± 0.29	**0.019**
LV EDV (mL/kg)	2.55 ± 0.19	1.83 ± 0.13	**0.026**
LV ESV (mL/kg)	0.96 ± 0.11	0.63 ± 0.08	0.060
LV SV (mL/kg)	1.59 ± 0.20	1.20 ± 0.07	0.161
LV EF (%)	62 ± 5	66 ± 2	0.522
LVIDd (cm)	3.20 ± 0.04	2.49 ± 0.08	**0.001**
LVIDs (cm)	2.24 ± 0.09	1.54 ± 0.06	**0.001**
LVPWd (cm)	0.78 ± 0.06	0.89 ± 0.07	0.273
LVPWs (cm)	1.09 ± 0.07	1.18 ± 0.08	0.457
IVSd (cm)	0.89 ± 0.04	0.75 ± 0.04	**0.041**
IVSs (cm)	1.16 ± 0.08	1.01 ± 0.03	0.142
PV peak V (m/s)	0.90 ± 0.10	0.79 ± 0.09	0.449
PV gradient (mmHg)	3.4 ± 0.8	2.6 ± 0.6	0.470
PR peak V (m/s)	0	0.21 ± 0.07	0.061
PR gradient (mmHg)	0	0.2 ± 0.1	0.078
TR peak V (m/s)	0	2.64 ± 0.27	**0.002**
TR gradient (mmHg)	0	28.8 ± 6.2	**0.019**
Ao peak V (m/s)	1.04 ± 0.15	0.73 ± 0.07	0.148
Ao gradient (mmHg)	4.58 ± 1.38	2.17 ± 0.43	0.198
MV E (m/s)	0.71 ± 0.06	0.57 ± 0.03	0.121
MV A (m/s)	0.41 ± 0.07	0.37 ± 0.06	0.654
MV E/A	1.81 ± 0.20	1.71 ± 0.27	0.787

*ECHO, echocardiography; bpm, beats per minute; Ao, aorta; PA, pulmonary artery; RV, right ventricle; PEP, pre-ejection period; AT, acceleration time; ET, ejection time; LA, left atrium; LV, left ventricle; EDV, end-diastolic volume; ESV, end-systolic volume; SV, stroke volume; EF, ejection fraction; IDd, inner-diameter at diastole; IDs, inner-diameter at systole; PWd, posterior wall at diastole; PWs, posterior wall at systole; IVSd, interventricular septum thickness at diastole; IVSs, interventricular septum thickness at systole; PV, pulmonic valve; V, velocity; PR, pulmonic regurgitation; TR, tricuspid; MV, mitral valve. Bold indicates p < 0.05*.

### RV Afterload

**Figure 4:**
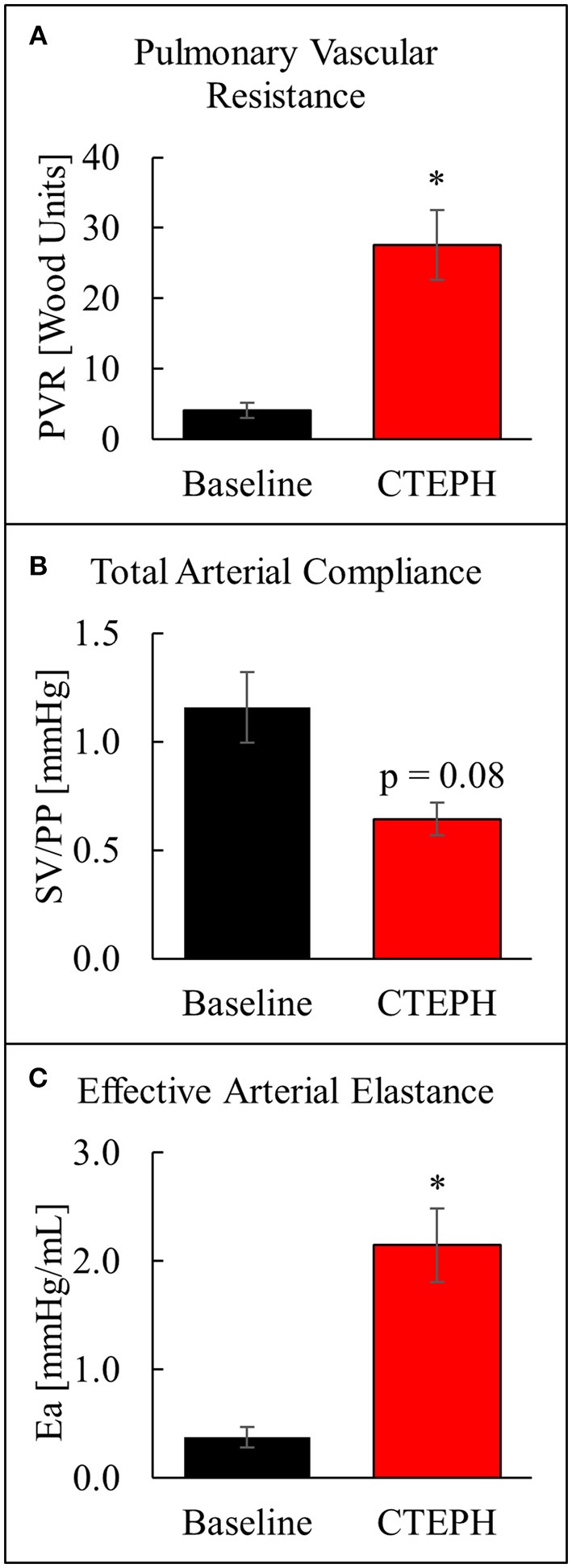
Changes in arterial properties as described by **(A)** pulmonary vascular resistance, **(B)** total arterial compliance, and **(C)** effective arterial elastance (*p < 0.05).

The chronic injection of microspheres caused an almost seven-fold increase in PVR (4.1 ± 1.1 v. 27.6 ± 5.0 [Wood units]; *p* = 0.022) (Figure 4A). It also caused an approximately 45% reduction in the total arterial compliance (1.16 ± 0.16 v. 0.64 ± 0.07 [mmHg]; *p* = 0.08) (Figure 4B). The increase in PVR and decrease in compliance resulted in a four-fold increase in E_a_ (0.38 ± 0.09 v. 2.15 ± 0.34 [mmHg/mL]; *p* = 0.012) (Figure 4C).

**Figure 5:**
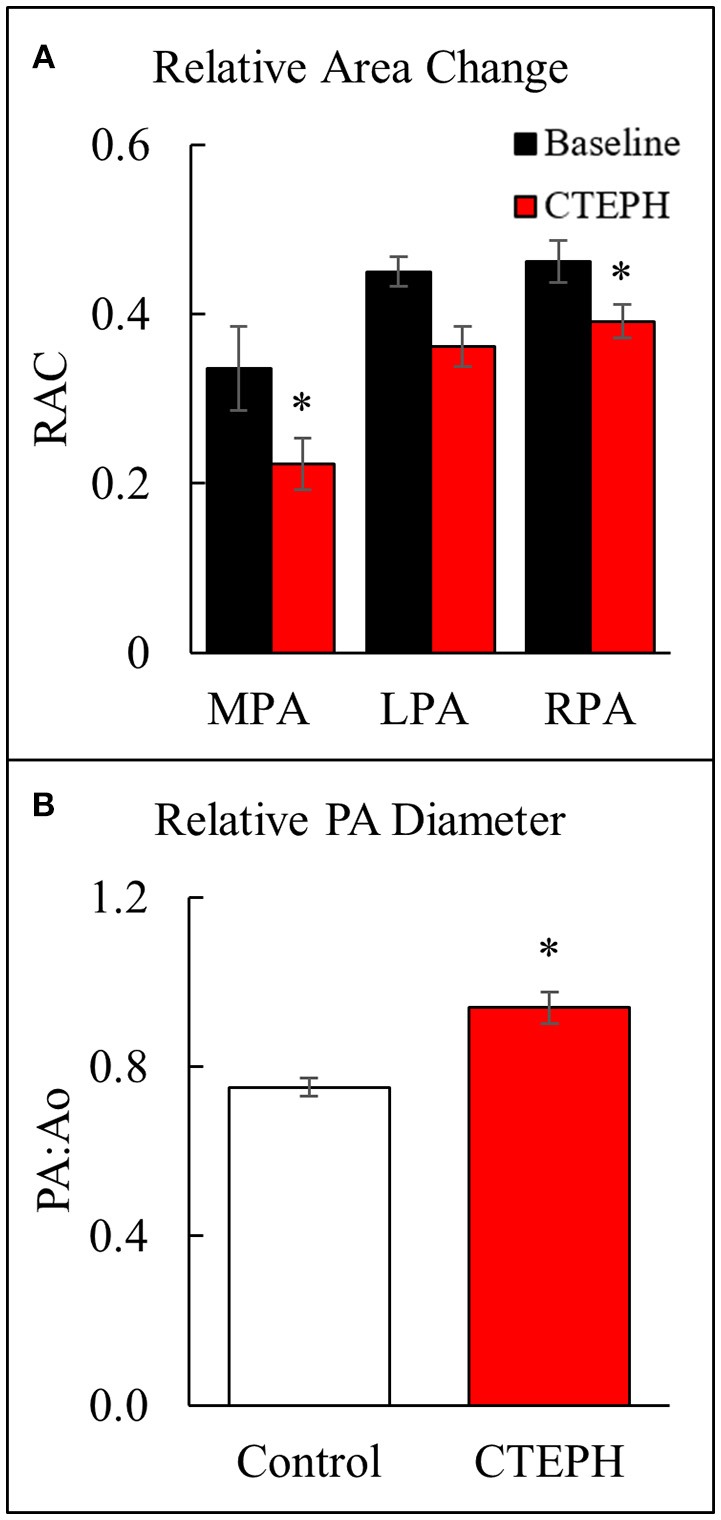
Changes observed in the PA. **(A)** The relative area change in the MPA, LPA, and RPA before and after chronic embolization as measured from MRI, and **(B)** The relative PA diameter normalized to the aortic diameter as measured from echo (*p < 0.05).

Proximal artery stiffening was assessed non-invasively using the RAC of the main, left, and right pulmonary arteries (MPA, LPA, and RPA, respectively) calculated from the MRI images. The RAC of the MPA was decreased by approximately 45% and the RPA by approximately 26% (Figure 5A). In addition, the maximal diameter of the PA relative to the maximal diameter of the aorta was significantly higher in the CTEPH animals, demonstrating PA dilation (Figure 5B).

**Figure 6:**
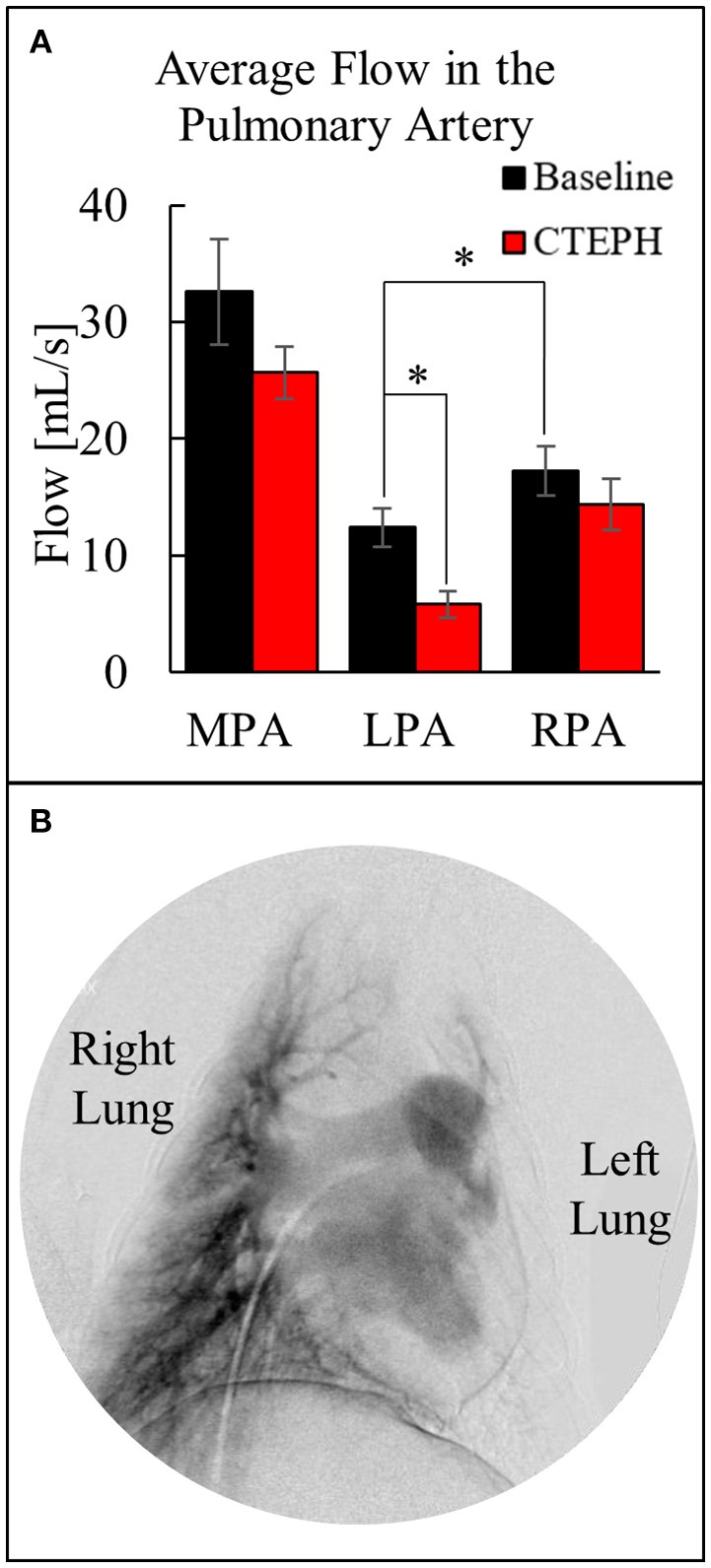
**(A)** Quantification of the average flow in the MPA, LPA, and RPA before and after chronic embolization as determined from MRI, and **(B)** Digital subtraction angiography image from a canine with CTEPH; very little perfusion in the left lung compared to the right lung (*p < 0.05).

The average blood flow was also calculated utilizing the two-dimensional phase contrast images of the PA, which decreased through the MPA after chronic embolization (Figure 6A). Despite there being significantly more flow in the RPA before embolization, more emboli were delivered to the left lung, resulting in a significant decrease in perfusion at the end of the study. This was confirmed by digital subtraction angiography (Figure 6B) and necropsy.

### RV Function

**Figure 7:**
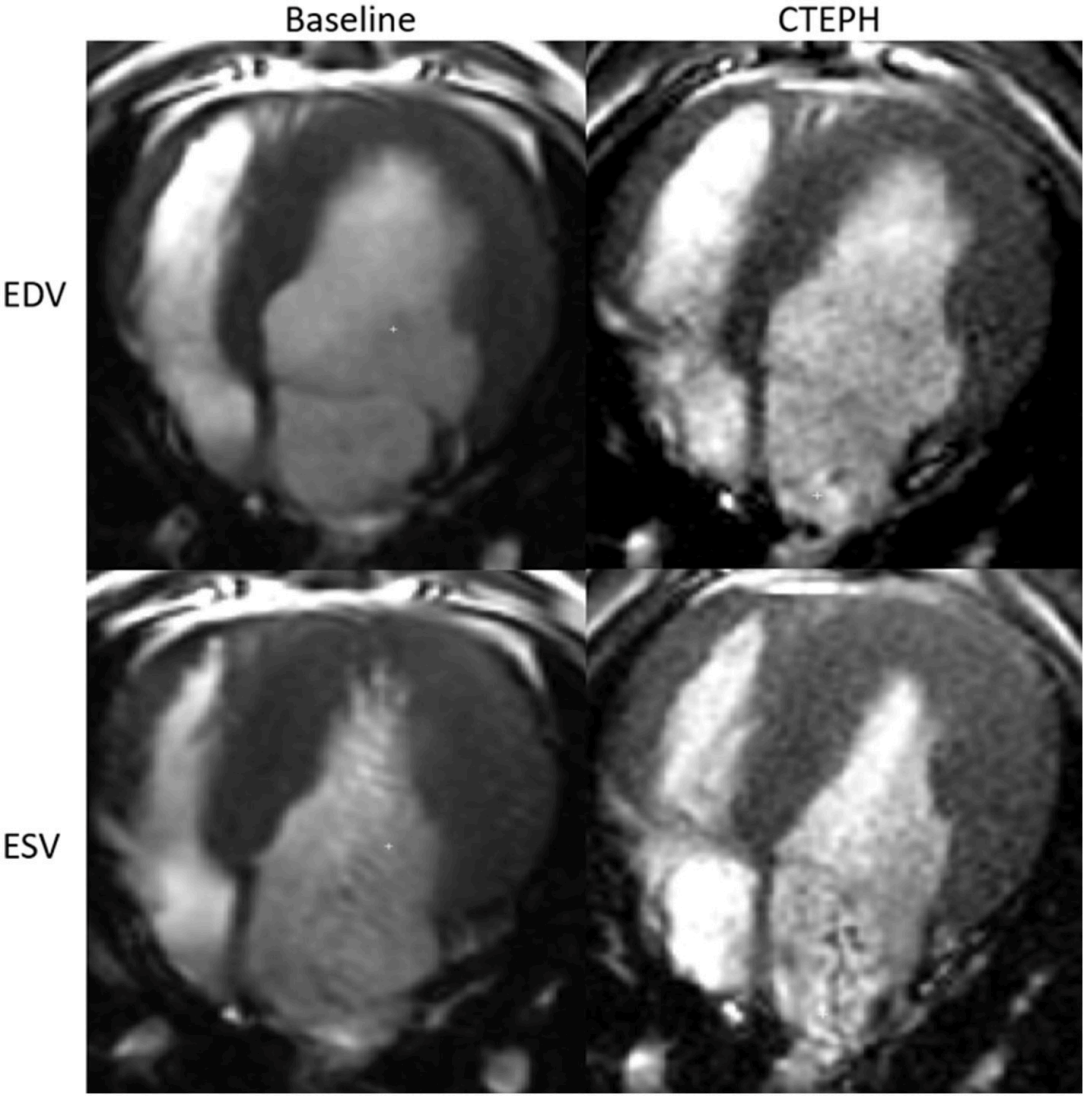
Representative MR images at end-diastole and end-systole for baseline and at the terminal end-point of CTEPH.

**Figure 8:**
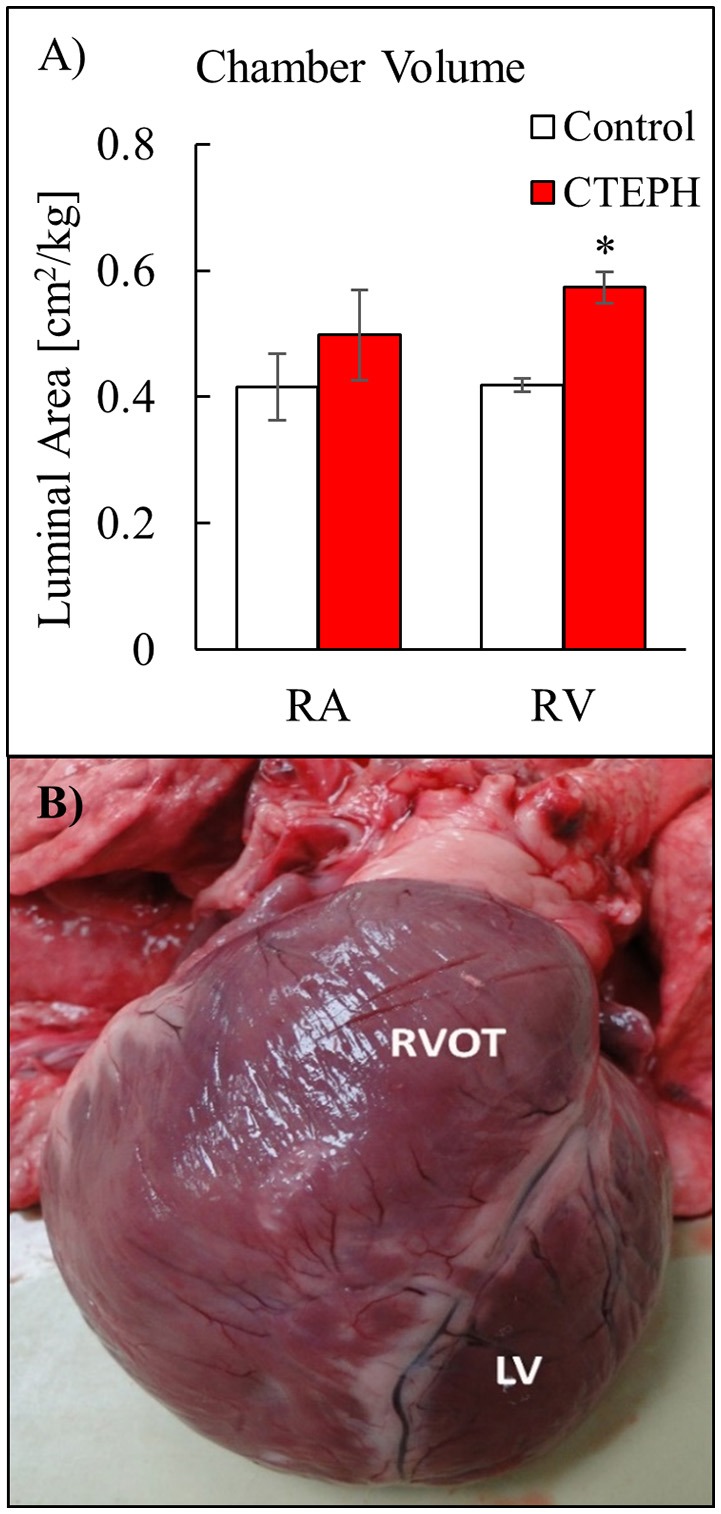
**(A)** Chamber volumes of the right heart from echo measurements, and **(B)** severe RV dilation in a dog with severe CTEPH. Anterior view comparing the dilated right ventricular outflow tract (RVOT) to the normal LV (*p < 0.05).

**Figure 9:**
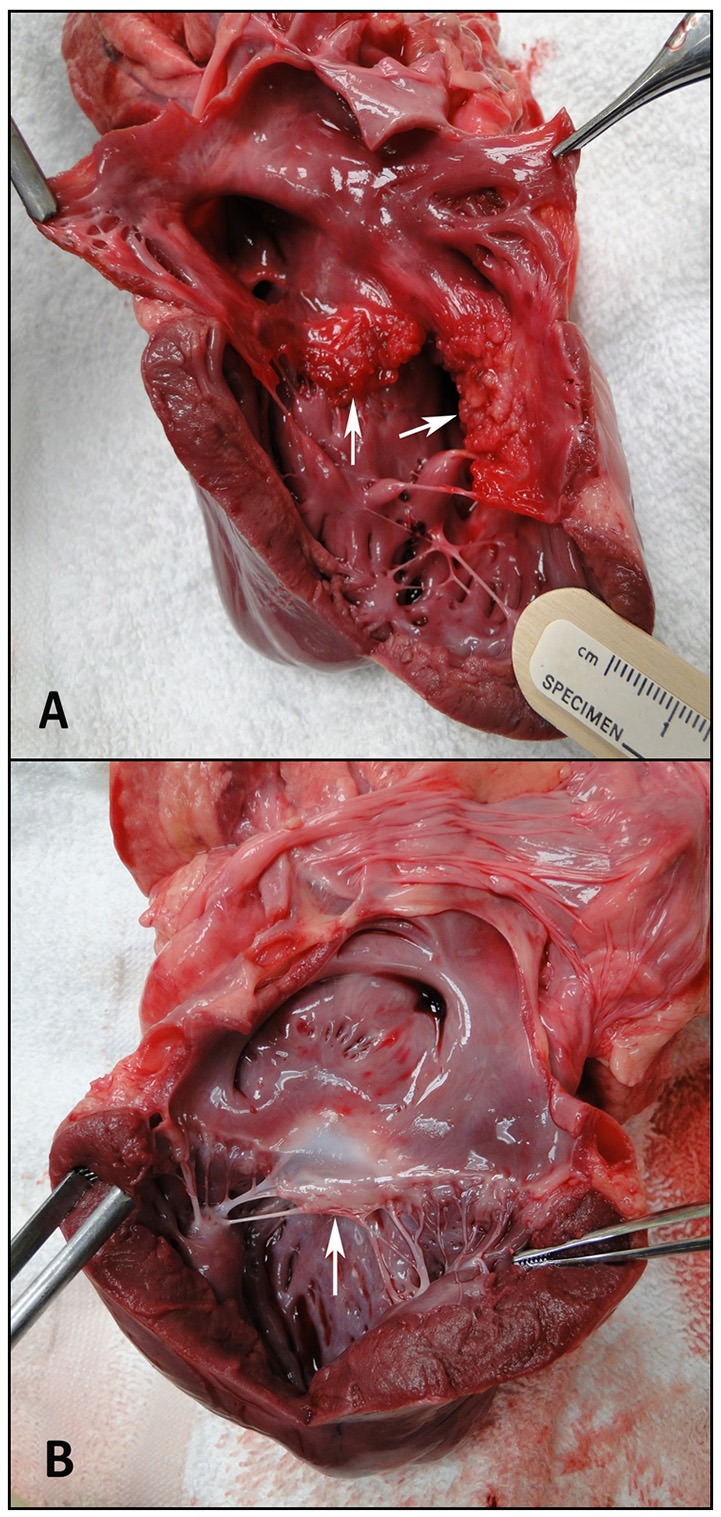
Visual inspection of the ventricles. **(A)** Tricuspid endocarditis. The arrows are showing the septal and mural tricuspid valve leaflets, and **(B)** a normal anterior mitral valve leaflet, shown by the arrow.

RV volumes calculated from the MRI contours revealed significant increases in the RV end-diastolic and end-systolic volumes (Table 2). Figure 7 shows representative MRI images in the same canine at end-diastole and end-systole for baseline and CTEPH measurements. These findings were supported by echo measurements (Figure 8A). Dilation of the RV outflow tract was visually apparent following tissue harvest (Figure 8B). It is noteworthy that tricuspid valve vegetative endocarditis, evident by echo and at necropsy by visual inspection (Figure 9), developed in all CTEPH animals.

**Figure 10:**
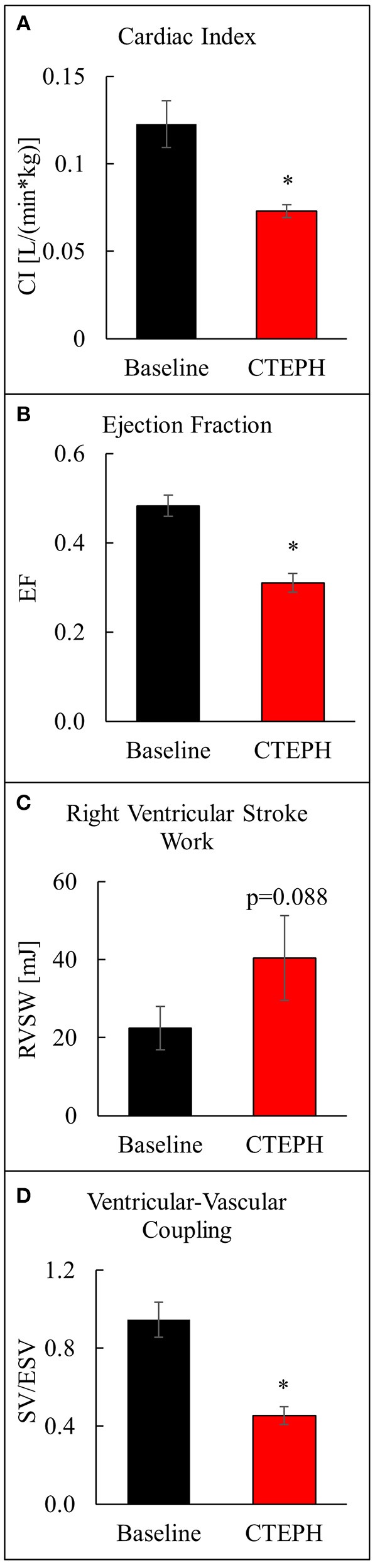
Assessment of RV function as described by **(A)** cardiac index, **(B)** ejection fraction, **(C)** right ventricular stroke work, and **(D)** ventricular-vascular coupling ratio (*p < 0.05).

**Figure 11:**
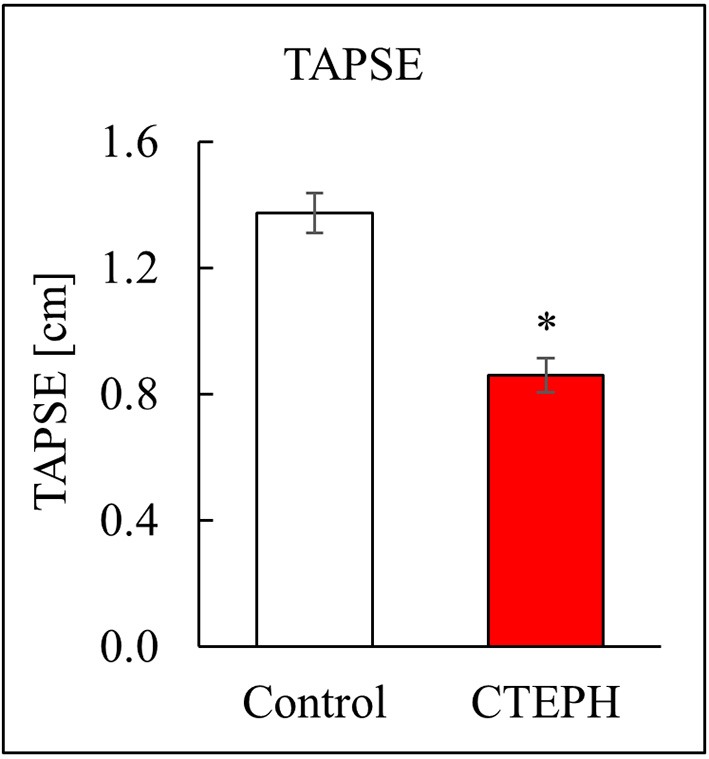
RV function as described by TAPSE, measured via echo (*p < 0.05).

CTEPH also caused RV failure, as evidenced by a 40% reduction in the CI (Figure 10A), a 36% reduction in EF (Figure 10B), and 80% increase in RVSW (Figure 10C) and significant ventricular-vascular uncoupling (Figure 10D). Moreover, by echo, CTEPH animals had significantly longer RV ejection times (Table 3) and decreased tricuspid annular plane systolic excursion (TAPSE) (Figure 11), which is often used as a clinical metric of RV function (33).

## Discussion

The lack of small and large animal models that recapitulate the key features of clinical CTEPH has impeded progress on successful, early diagnostics and greatly limited the available therapeutic and pharmaceutical treatment options. Many investigators have attempted to create large animal models of CTEPH utilizing various approaches with varying success, but few have successfully captured the clinical endpoint—RV failure. In this present study, we replicated this hallmark feature of clinical CTEPH in a canine model utilizing only the recurring injections of microspheres.

Contrary to several other studies where microspheres alone did not sufficiently invoke a hemodynamic or histological response (15, 25), we showed its feasibility to induce RV failure in a large animal model of CTEPH as evidenced by a significantly reduced CI, EF, and TAPSE. The advantages of the microsphere models are the ease of delivery, comparatively less-invasive surgical procedures, and the ability to generate CTEPH in both lungs. Failure of previous models is most likely due to the lack of consensus on the optimal embolic material, size, and delivery frequency, as well as the overall time necessary to achieve functional or structural changes. While we have shown the capability of this technique, one of the main disadvantages is the duration necessary to achieve these results. It required 6 months, on average (range 4–8 months), before sufficient levels of PH were observed, which is much longer than most studies have attempted (3, 18, 20, 21, 25).

An extended induction phase does have some advantage as it is notably closer to the rate of disease progression seen in humans (10), and unlike acute studies, allows for RV remodeling. RV remodeling was determined by RV enlargement as assessed by MRI, echo, and visual inspection following the study. While RV remodeling can be beneficial, we speculate that these changes were already maladaptive as systolic function had declined and VVC was shown to have decreased to approximately 0.45, signifying severe uncoupling.

While not the focus of this study, this model could offer the opportunity to study the mechanistic progression of CTEPH development. Optimization of the embolic delivery may allow for insight into the vascular response and adaptation occurring between each embolization, as well as how these alterations lead to RV remodeling, the transition from adaptive to maladaptive RV remodeling, as well as mechanistic sex differences. A better understanding of this progression could lead to earlier diagnostic markers and better treatment options.

Digital subtraction angiography images showed that there was an uneven distribution of microspheres delivered to the LPA compared to the RPA, which was visually confirmed at necropsy. Since the RPA typically has more flow, we believe this is due to the tip of the catheter being directed more toward the LPA, causing a disproportionate number of beads to be delivered. If the catheter tip was placed more proximally, we speculate that there would have been a significant decrease in flow through the RPA between baseline and CTEPH measurements, and the flow distribution would be more equal across both lungs. Furthermore, two catheters could be utilized and positioned such that the microsphere distribution between the two lungs could be delivered as desired.

Limitations of this study include the asynchronous acquisition of pressures, volumes, and cardiac output, the variable induction time for CTEPH development, as well as the lack of histological analysis from the RV. In addition, PH is associated with increased anesthesia risk so several of the animals were given a dose of atropine, an anti-muscarinic agent that directly increases heart rate by decreasing the parasympathetic tone on the sinoatrial node (34). The half-life of atropine is relatively short, so we do not suspect that this agent had any substantial influence on our other metrics of interest such as RV volumes. Phase contract MRI has been shown to underestimate flow with turbulent stenotic jets, which can cause significant signal loss due to intravoxel dephasing (35). This is one possible explanation for the increase in flow mismatch between RPA+LPA flow and MPA flow that occurred with the CTEPH animals. Lastly, having an indwelling catheter within the heart for that length of time created several challenges. One such challenge was the increased risk of infection and sepsis. The canines were closely monitored for fever, distress, and other signs of infection, and given antibiotics as recommended by veterinary staff. Three of the four dogs also dislodged their indwelling catheter, requiring an additional invasive procedure to secure another one in place for microsphere delivery and pressure monitoring. We also believe the catheter contributed to the development of vegetative endocarditis that not only led to tricuspid regurgitation, but also severely limited catheter access during the terminal procedure and prevented our complete RHC and hemodynamic studies. Lastly, the tricuspid regurgitation, and to a lesser extent the pulmonic regurgitation, adds uncertainty to our measurement of SV.

In conclusion, CTEPH was induced in a canine model using repeated injections of microspheres into the PA via an indwelling catheter, successfully inducing RV failure and RV remodeling, which has not been observed in previous acute embolization models (27). Since the progression of pulmonary vascular pathology to RV failure is still poorly understood, this large animal model could provide valuable insight into disease progression. The recapitulation of heart failure phenotypes in large animals could provide critical links for therapeutic and pathophysiologic intervention in clinical practices, and warrants further study.

## Author Contributions

NC, CF, TH, MB, HK, and OF designed the study. HK, TH, and OF collected the data and managed animal care. AM, HK, and NC contributed to data analysis and interpretation. AM wrote the manuscript. All authors reviewed and approved the manuscript.

### Conflict of Interest Statement

The authors declare that the research was conducted in the absence of any commercial or financial relationships that could be construed as a potential conflict of interest.
